# The complete chloroplast genome of *Torilis scabra* (Apiaceae)

**DOI:** 10.1080/23802359.2019.1661303

**Published:** 2019-09-05

**Authors:** Xue-Ying Yao, Zhi-Xiang Chen, Qi-Zhi Wang

**Affiliations:** Department of Horticulture, Laboratory of Floral Resources, Huaqiao University, Xiamen, P. R. China

**Keywords:** *Torilis scabra* (Thunb.) DC., complete chloroplast genome, Apiaceae

## Abstract

*Torilis scabra* (Thunb.) DC. is widely distributed in China and Japan and has been introduced to North America. In this study, the complete chloroplast genome sequence of the *T*. *scabra* was obtained by de novo assembly using the NGS data. The chloroplast genome of *T. scabra* was 157,855 bp in length and divided into four distinct regions, such as large single-copy region (85,362 bp), small single-copy region (17,993 bp), and a pair of inverted repeat regions (27,250 bp). The genome annotation predicted a total of 127 genes, including 82 protein-coding genes, 37 tRNA genes, and 8 rRNA genes. Phylogenetic analysis with reported chloroplast genomes showed that *T. scabra* has a close genetic relationship with *Anthriscus cerefolium*.

*Torilis scabra* (Thunb.) DC. is an annual or perennial herb that belongs to the family Apiaceae. It usually grows in mixed forests on mountain slopes or in valleys, roadsides, especially in disturbed areas on hillsides at an altitude of 250–2400 meters. *Torilis scabra* is widely distributed in China and has reputed medicinal value (She et al. 2005). The fruit shape of *Torilis* is similar to other genus of the Apiaceae, such as the *Sanicula* (Chen et al. 2018). Some confusion on identification exists within the *Torilis* genus with similar species frequently misidentified in herbaria and the literature (Antonio et al. 2014). Thus, it shows that our understanding of this genus is still insufficient and more effective molecular methods are needed to determine the phylogenetic relationships. The chloroplast genome structure is relatively conservative and the base mutation rate is moderate, which has been widely used phylogenetic study for various plant groups (Li et al. 2017). Here, we first report the complete chloroplast genome of *T. scabra*, which provides more data for the phylogenetic and evolutionary relationships of this genus.

The fresh and healthy leaves of *T. scabra* were collected from Yongchun county (25°26′12′′N 117°53′27′′E), Fujian Province, China. Voucher specimens were deposited in Hiaoqiao University Herbarium (00004531). Total genomic DNA was extracted by Plant Genomic DNA Kit (Sangon Biotech, Shanghai, China). Paired-end reads were sequenced by using Illumina Hiseq Platform (Illumina, San Diego, CA, USA). Approximately, 10 Gb of paired-end (150 bp) sequence data were randomly extracted from the total sequencing output and used as input for NOVOPlasty (Dierckxsens et al. 2017) to assemble the plastid genome. The plastid genome of *Pterygopleurum neurophyllum* (GenBank accession number: NC_033345) was used as the seed sequence. The genes in chloroplast genome were predicted using Geneious version 11.0.4 (Kearse et al. 2012) and corrected manually. The phylogenetic tree was constructed by maximum likelihood method with 1000 bootstrap replicates was inferred using MEGA7.0 (Kumar et al. 2016) from alignments created by the MAFFT (Katoh et al. 2002) using plastid genomes of 15 species.

The complete chloroplast genome of *T. scabra* (GenBank accession number: MN105615) was 157,855 bp in total sequence length, which was separated into four distinct regions such as large single-copy (LSC) region was 85,362 bp, small single-copy (SSC) region was 17,993 bp, and a pair of inverted repeat regions are 27,250 bp in each length. Overall, GC contents of *T. scabra* chloroplast genomes were 37.40%. The chloroplast genome detected a total of 127 genes including 82 protein-coding genes, 37 tRNA genes, and 8 rRNA genes.

To determine the phylogenetic position of *T. scabra*, a phylogenetic analysis was carried out among 13 complete chloroplast genomes of Apiaceae from NCBI. The phylogenetic analysis showed that *T. scabra* is closely related to *A. cerefolium* and is distinct from *S. chinensis* (Figure 1). This complete chloroplast genome can provide new evidence for taxonomy and can be further used for population genomic studies, genetic engineering research in *Torilis*.

**Figure 1.**
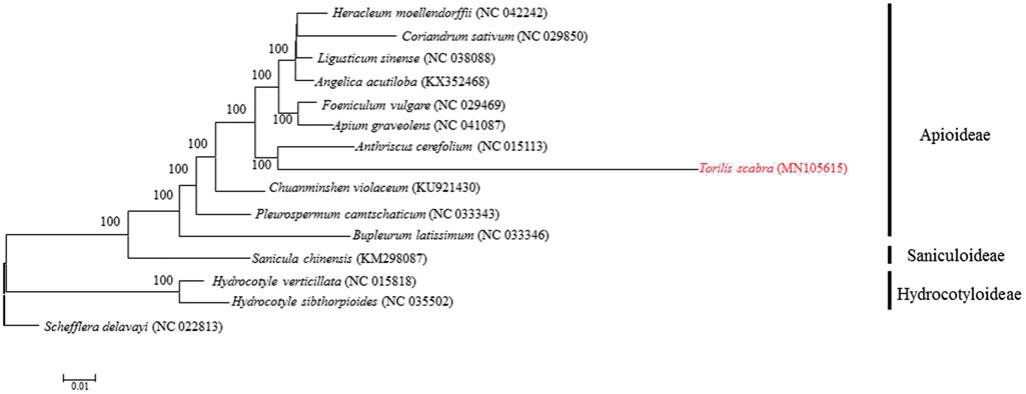
ML phylogenetic tree of *Torilis scabra* with other 13 species in the Apiaceae order was constructed by chloroplast genome sequences. Numbers on the nodes are bootstrap values from 1000 replicates. *Schefflera delavayi* was selected as an outgroup.
